# Impact of Alien Plant Invaders on Pollination Networks in Two Archipelagos

**DOI:** 10.1371/journal.pone.0006275

**Published:** 2009-07-17

**Authors:** Benigno Padrón, Anna Traveset, Tine Biedenweg, Diana Díaz, Manuel Nogales, Jens M. Olesen

**Affiliations:** 1 Institut Mediterrani d'Estudis Avançats (CSIC-UIB), Esporles, Mallorca, Balearic Islands, Spain; 2 Island Ecology and Evolution Research Group (CSIC-IPNA), La Laguna, Tenerife, Canary Islands, Spain; 3 Department of Biological Sciences, Aarhus University, Aarhus, Denmark; University of Zurich, Switzerland

## Abstract

Mutualistic interactions between plants and animals promote integration of invasive species into native communities. In turn, the integrated invaders may alter existing patterns of mutualistic interactions. Here we simultaneously map in detail effects of invaders on parameters describing the topology of both plant-pollinator (bi-modal) and plant-plant (uni-modal) networks. We focus on the invader *Opuntia* spp., a cosmopolitan alien cactus. We compare two island systems: Tenerife (Canary Islands) and Menorca (Balearic Islands). *Opuntia* was found to modify the number of links between plants and pollinators, and was integrated into the new communities via the most generalist pollinators, but did not affect the general network pattern. The plant uni-modal networks showed disassortative linkage, i.e. species with many links tended to connect to species with few links. Thus, by linking to generalist natives, *Opuntia* remained peripheral to network topology, and this is probably why native network properties were not affected at least in one of the islands. We conclude that the network analytical approach is indeed a valuable tool to evaluate the effect of invaders on native communities.

## Introduction

A growing number of studies show that mutualistic interactions between plants and animals promote the integration of invasive species into native communities and can actually influence the dynamics and ultimate success of many plant and animal invasions [1]–[3]. In turn, once integrated, the invader may dramatically alter the mutualistic interaction structure, with negative consequences for the persistence of native species (see examples in [4]). In natural systems with a nested interaction structure, such as plant-pollinator networks [5], the impact of an alien may rapidly cascade out through the entire network because all species are closely linked to each other [6]. Thus, the importance of aliens at the level of network is expected to be pronounced. However, the exploration of this aspect of invasion biology is relatively young (for alien plants [1], [7], [8] and for alien pollinators [3]). The study of native-alien mutualisms at community level and within the framework of ecological networks is promising as it provides new insights into the process of integration of aliens alongside their impact on the native communities, and it may be used to improve our predictions about effects of biological invasions.

A network approach is especially urgent in island ecosystems, given their high rate of invasions [4], [9], [10]. In many islands, entire sets of pollinators (and also seed dispersers) are disappearing due to alien invaders (e.g. [11], [12]) with negative consequences for most plants that depend upon them for regeneration [4]. Island studies have reported high network connectance and the presence of super-generalist species, both among plants and pollinators [1]. Connectance is the fraction of possible links that are realized. Super-generalists are the most linked species in pollination and seed-dispersal networks. These highly connected species tend to be endemic, and seem to be the most likely promoters of alien integration [1], [13].

We investigated the impact of the widespread alien genus *Opuntia* on native plant-pollinator communities in the Canary Islands and the Balearic Islands. We compared native communities invaded by *Opuntia* with adjacent non-invaded communities, with the main goal of testing if this alien modifies the topology of the native network. We use a set of characterizing parameters previously used in the analysis of mutualistic networks [5], [6], [14] as well as others used in the analysis of other network types [15]. Specifically, for the analyses of uni-modal networks, we used different measures of *centrality* (a parameter that tells us how central a species is with respect to the entire network and to what extent it connects different regions of the network) and *centralization* (a centrality measure for the entire network) [16], which can help us to evaluate the role of the aliens in the community and the possible changes these may have to the native species. The topology of both bi-modal (plant-pollinator) and uni-modal (plant-plant) networks are examined simultaneously. The former describes trophic and reproductive interactions between a community of flowering plants and a community of pollinator species within a well-defined habitat, whereas the latter describes interactions between plants (plants are linked to each other if they share pollinators). Uni-modal networks are a useful tool in the study of competition or facilitation among plants for pollinators. Since bi-modal and uni-modal networks map different types of interactions in a community, the information they provide is complementary and can help us to understand in detail how an alien species influences the receiving system. In this work about a plant invader, we do not consider the pollinator uni-modal network.

Mostly based on previous information, we make the following predictions:

1. The integration of an alien plant into a pollination network is mediated by the most generalist (native or alien) pollinators [1], [3], [7], [13] and *Opuntia* interacts most frequently with generalist insects (super-generalists *sensu* [1]).
2. Network connectance is not influenced by the presence of the alien, despite the fact that the frequency of pollinator visits can be altered. Links (documented by at least one interaction in presence/absence matrices) rarely disappear completely even when alien species compete heavily with natives for pollinators but their strength often weakens.
3. Average linkage level for plants in the community increases if the alien plant promotes facilitation over competition for pollinators (i.e. if it mediates new interactions between pollinators and native plants); likewise, linkage level for insects decreases if the alien competes with natives for pollinators.
4. Since *Opuntia* is not expected to link exclusively to native specialist pollinators, we anticipate that it causes an increase in network nestedness.
5. *Opuntia* may begin as a peripheral species with only 1–few links, and then as the invasion progresses become promoted to either a hub or a connector, i.e. a species linking different subgroups of the network. Both situations may affect centrality and centralization, and consequently overall network topology, at the current point in the invasion.
6. Finally, and taking advantage of our study design (4 sites, 2 years) we expect a turnover in species composition within-network but also a constancy in the general patterns describing network topology, as previously reported in other studies [17]–[19].

## Results

A total of 2330 and 1047 insect flower visits were recorded on Menorca and Tenerife, respectively. The number of insect species on each island network was, coincidently, the same (n = 59). They visited 16 plant species from 15 families in Menorca and 11 plant species from nine families in Tenerife. The proportion of species in the different insect orders differed greatly between the two islands: Menorca had 35.6% Hymenoptera, 25.4% Diptera, 20.3% Coleoptera, 13.6% Lepidoptera and 5.1% others, whereas Tenerife had 39.0% Hymenoptera, 50.8% Diptera, 5.1% Coleoptera, 1.7% Lepidoptera and 3.4% others. *Opuntia maxima* in Menorca behaved as a hub in the network, being one of the most generalist plants in the community; it was visited mainly by the most abundant and generalist pollinators, such as *Apis mellifera*, *Bombus terrestris*, *Gonepteryx cleopatra*, *Halictus scabiosa*, *Oedemera* sp., *Oxythyrea funesta*, *Rhodanthidium septemdentatum*, *Stenopleurus* sp. and *Xylocopa violacea*. In Tenerife, by contrast, *O. dillenii* was a specialist, being visited only by a few species; these species, however, were mainly the most abundant and generalist pollinators: *Apis mellifera*, *Bombus canariensis* and *Lasioglossum viride*. For both islands, thus, we confirmed the first prediction that *Opuntia* is integrated by the most generalist insects in the receiving communities.

### Plant-pollinator networks

**Figure 1:**
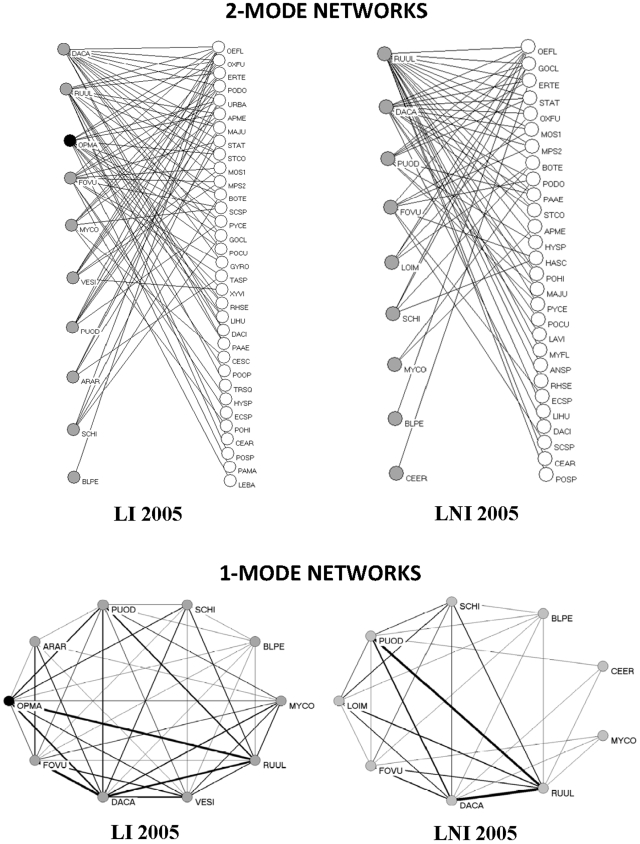
Representation of the bi-modal and uni-modal networks for Llimpa (Menorca; data from 2005). Plant species are shown as grey nodes and plant visitors as white nodes. *Opuntia* is the only black-coloured node. Links are shown as black lines connecting plants and pollinators. In the uni-modal networks, line thickness is proportional to number of insect species shared by plants. Note: LI, Invaded Llimpa site; LNI, Non-Invaded Llimpa site.

Mainly because of the few co-flowering plant species with *Opuntia*, network size was rather small in both islands (Table 1, Figure 1). Network size is known to influence some of the parameters describing network topology; we thus controlled for that when comparing the impact of the alien species on the different parameters across localities. In accordance with our second prediction, we found that the presence of the alien in the networks did not influence connectance (*C*) in either island (Table 2).

**Table 1:** Parameters for the bi-modal and uni-modal networks.

Island	Locality	Site	2-mode networks												
			P	A	S	M	I	C	<L_n_>	<L_m_>	MN		N*	BR	
Tenerife	Ravine	I 2005	11	20	31	220	43	0.195	3.91±2.63	2.15±1.23	0.69	ns	0.14	23	ns
		NI 2005	10	21	31	210	47	0.224	4.70±2.00	2.24±1.73	0.66	ns	0.16	21	ns
	Windmill	I 2005	6	17	23	102	24	0.235	4.00±2.83	1.41±0.87	0.70	ns	0.22	11	ns
		NI 2005	5	14	19	70	19	0.271	3.80±2.59	1.36±0.84	0.74	ns	0.18	8	ns
	Ravine	I 2006	7	32	39	224	51	0.228	7.29±5.65	1.59±0.91	0.73	*	0.29	24	ns
		NI 2006	5	29	34	145	40	0.276	8.00±5.29	1.38±0.62	0.65	ns	0.20	19	ns
Menorca	Llimpa	I 2005	10	33	43	330	86	0.261	8.60±5.25	2.61±1.64	0.78	*	0.34	36	ns
		NI 2005	9	28	37	252	62	0.246	6.89±6.72	2.21±1.42	0.87	*	0.31	21	ns
	Cardona	I 2005	8	29	37	232	65	0.280	8.13±4.32	2.24±1.35	0.72	*	0.30	27	ns
		NI 2005	6	16	22	96	31	0.323	5.17±4.22	1.94±1.00	0.69	ns	0.02	9	ns
	Llimpa	I 2006	9	43	52	387	104	0.269	11.56±10.31	2.42±1.18	0.78	*	0.32	33	ns
		NI 2006	7	29	36	203	59	0.291	8.43±6.13	2.03±1.24	0.77	*	0.27	23	ns

Island	Locality	Site	1-mode networks													
			N	m	d	<l>	D	<c>	<dc>	<cc>	<bc>	DC	CC	BC	CCO	
Tenerife	Ravine	I 2005	11	24	0.436	1.69	3	0.68±0.31	4.36±2.29	0.62±0.13	0.077±0.097	0.44	0.50	0.20	0.25	ns
		NI 2005	10	34	0.756	1.24	2	0.86±0.10	6.80±1.75	0.82±0.12	0.031±0.036	0.31	0.43	0.10	0.68	*
	Windmill	I 2005	6	7	0.467	1.30	2	0.70±0.45	2.33±1.51	0.55±0.29	0.050±0.122	0.50	—	0.30	0.89	*
		NI 2005	5	6	0.600	1.00	1	1.00±0.00	2.40±1.34	0.64±0.36	0.000±0.000	0.25	—	0.00	—	
	Ravine	I 2006	7	15	0.714	1.29	2	0.86±0.19	4.29±1.50	0.80±0.16	0.057±0.084	0.40	0.51	0.14	0.98	*
		NI 2006	5	6	0.600	1.40	2	0.60±0.43	2.40±1.14	0.74±0.17	0.133±0.254	0.67	0.76	0.56	0.99	*
Menorca	Llimpa	I 2005	10	40	0.899	1.11	2	0.92±0.06	8.00±1.15	0.91±0.10	0.014±0.012	0.14	0.21	0.01	0.97	*
		NI 2005	9	27	0.750	1.25	2	0.89±0.14	6.00±1.87	0.82±0.14	0.036±0.048	0.32	0.43	0.09	0.91	*
	Cardona	I 2005	8	26	0.929	1.07	2	0.94±0.05	6.50±0.76	0.94±0.09	0.012±0.010	0.10	0.15	0.01	0.96	*
		NI 2005	6	11	0.733	1.27	2	0.77±0.41	3.67±1.37	0.81±0.14	0.067±0.163	0.40	0.50	0.40	0.82	*
	Llimpa	I 2006	9	28	0.778	1.22	2	0.88±0.10	6.22±1.64	0.84±0.12	0.032±0.035	0.29	0.40	0.09	0.82	*
		NI 2006	7	18	0.857	1.14	2	0.91±0.11	5.14±1.07	0.89±0.12	0.029±0.036	0.20	0.28	0.04	0.88	*

**Note**: Abbreviations: **P**, number of plant species; **A**, number of animals species; **S** (species richness) = A+P; **M** (network size) = A*P; **I**, number of links in 2-mode networks; **C** (connectance) = I/M; **<L_n_>**, average number of links per plant species and standard deviation; **<L_m_>**, average number of links per pollination species and standard deviation; **MN**, matrix nestedness using Aninhado program; N*, relative matrix nestedness (see text for more explanations); **BR**, Brualdi and Sanderson index; *, *P*<0.05; **ns**, non-significance; **N**, community size; **m**, number of links between N; **d** (link density) = 2*m/(N*(N−1)); **<l>**, average shortest path length, i.e. average shortest distance among any pair of species; **D**, diameter, i.e. longest shortest path among any pair of species; **<c>**, average clustering coefficient, i.e. link density among neighbours to a species (X±SD); **<dc>**, degree centrality (X±SD); **<cc>**,closeness centrality (X±SD); **<bc>**,betweenness centrality (X±SD); **DC**, degree centralization; **CC**, closeness centralization; **BC**, betweenness centralization; **CCO**, connectivity correlation (R^2^); *, *P*<0.05; **ns**, non-significance. All values are absolute, i.e. not corrected by network size.

**Table 2:** Results of Wilcoxon's tests analysing differences in the bi-modal and uni-modal network parameters.

*alien effects*		invaded sites		non-invaded sites		Z	*P*
		mean	SD	mean	SD		
**2-mode**	C (connectance)	0.0013	0.0007	0.0023	0.0015	−1.826	0.068
	N* (relative nestedness)	0.2489	0.0872	0.1682	0.1178	−1.461	0.144
	BR (Brualdi & Sanderson index)	0.1364	0.0993	0.0988	0.0213	−0.730	0.465
**1-mode**	d (link density)	0.0806	0.0317	0.1003	0.0243	−1.461	0.144
	DC (degree centralization)	0.2950	0.2042	0.3200	0.0616	−0.365	0.715
	CC (closeness centralization)	0.2867	0.1872	0.4533	0.0404	−1.069	0.285
	BC (betweeness centralization)	0.1300	0.1445	0.1475	0.1742	0.000	1.000

*year-to-year variation*		2005		2006		Z	*P*
		mean	SD	mean	SD		
**2-mode**	C (conectance)	0.0009	0.0001	0.0012	0.0005	−1.461	0.144
	N* (relative nestedness)	0.2365	0.1016	0.2704	0.0534	−0.365	0.715
	BR (Brualdi & Sanderson index)	0.1042	0.0415	0.1146	0.0460	0.000	1.000
**1-mode**	d (link density)	0.0719	0.0221	0.1077	0.0169	−1.461	0.144
	DC (degree centralization)	0.3025	0.1234	0.3900	0.2038	−0.730	0.465
	CC (closeness centralization)	0.3925	0.1261	0.4875	0.2045	−1.095	0.273
	BC (betweeness centralization)	0.1000	0.0779	0.2075	0.2385	−0.730	0.465

Alien effects were tested using pairs of sites (invaded *vs.* non-invaded) observed in 2005. Year-to-year variation was observed comparing only the pairs of sites observed two consecutive seasons. *C*, *BR* and *d* have been corrected for network size when performing the analyses.

Within each island, there were differences among localities in linkage levels for both plants (*L_n_*) and insects (*L_m_*) (Table 3); they were significantly higher at Cardona in Menorca and at Windmill in Tenerife. Islands did not differ in *L_n_*, but pollinators on Tenerife had a higher *L_m_* than those from Menorca, i.e. insects interacted with a larger number of plants on the oceanic Tenerife. *Opuntia* did not significantly modify *L_n_* and this was consistent between localities within each island (Table 3). By contrast, average *L_m_* was lower in invaded sites, suggesting that the alien is attracting pollinators away from natives, although this was not consistent between localities within each island (Table 3). Our prediction on the impact of the alien on linkage levels is thus only partly confirmed; *Opuntia* does not modify the number of insect species that native plants interact with, but it can promote changes in the number of plants that insects visit.

**Table 3:** GLMs of parameters in the bi-modal and uni-modal networks.

			2-mode networks						1-mode networks								
			L_n_			L_m_			dc			cc			bc		
		df	log-likelihood	χ^2^	*P*	log-likelihood	χ^2^	*P*	log-likelihood	χ^2^	*P*	log-likelihood	χ^2^	*P*	log-likelihood	χ^2^	*P*
**alien effects**	intercept	1	164.960			650.6751			−2.612			16.990			72.198		
	island	1	165.275	0.630	0.427	652.9845	4.619	0.032	2.310	9.843	0.002	26.264	18.548	0.000	72.700	1.005	0.316
	locality(island)	2	172.106	13.662	0.001	666.5204	27.072	0.000	2.318	0.016	0.992	27.102	1.676	0.433	73.036	0.672	0.715
	invasion	1	172.802	1.393	0.238	670.6381	8.235	0.004	3.639	2.643	0.104	27.110	0.016	0.901	73.076	0.079	0.779
	locality(island*invasion)	3	173.390	1.176	0.759	677.3048	13.333	0.004	9.931	12.584	0.006	34.183	14.146	0.003	75.917	5.683	0.128
**year-to-year variation**	intercept	1	−32.374			959.617			−1.116			28.708			−23.202		
	year	1	−30.766	3.217	0.073	961.503	3.772	0.052	−1.086	0.059	0.809	29.152	0.887	0.346	−21.523	3.359	0.067
	invasion	1	−29.997	1.538	0.215	968.421	13.837	0.000	−0.883	0.406	0.524	29.748	1.191	0.275	−21.379	0.287	0.592
	year*site	1	−29.628	0.738	0.390	969.625	2.408	0.121	−0.503	0.760	0.383	30.012	0.530	0.467	−20.816	1.126	0.289

Alien effects tested using pairs of sites (invaded *vs.* non-invaded) observed in 2005. Year-to-year variation using pairs of sites observed two consecutive seasons.

Levels of nestedness varied among sites (Table 1). In Tenerife, the network was nested only at the invaded site at Ravine and only in 2006. By contrast, all networks in Menorca were nested, except for the non-invaded site at Cardona. Relative nestedness showed higher values in invaded than in non-invaded sites in five out of the six study pairs, albeit differences were not significant (Table 2). Brualdi indices did not differ significantly between invaded and non-invaded sites. The null model II (which considers that the probability of each cell being occupied depends on the total number of links of the column and the row defining the cell position) provided a more conservative test, giving lower significant levels than when using model I (each cell in the matrix has the same probability of being occupied) in all examined networks. Different software and algorithms have been proposed to estimate nestedness, and here we use three options. *Aninhado* and *Binmatnest* programs showed similar significance levels, although the latter gave lower temperature values (i.e. higher nestedness). The *Nestedness* software consistently produced non-significant results, which is attributed to the higher probability of making a type II error when testing matrices using a model similar to model II (cf. [20]). Thus, referring to our fourth prediction, although we found some evidence that invaded sites tend to be somewhat more nested in some localities than their paired non-invaded sites, we did not detect an overall significant effect of the invasion on this network parameter.

### Plant-plant networks

Table 1 shows all parameter values describing the uni-modal plant networks. At the species level, centrality measures (*dc*, *cc* and *bc*) varied between islands, although differences were significant only for *dc* and *cc* (Table 3), both being higher in Menorca than in Tenerife. This indicated that a higher number of plants in Menorca shared pollinators with other plants than in Tenerife and that such plants were more closely connected (with shorter paths between them). No significant differences were found between localities within each island for any of the three centrality measures, and no effect of the presence of *Opuntia* was detected in either case (Table 3). When examining path length (<l>) and clustering coefficient (<c>), we found that our networks showed low and hi0gh values, respectively (Table 1), which is consistent with previous findings in other uni-modal pollination networks [6]. Invaded and non-invaded sites did not differ significantly in either parameter (*Z* = 1.572, *P* = 0.116 for <l> and *Z* = 0.314, *P* = 0.753 for <c>). At the network level, the presence of *Opuntia* did not significantly influence any of the three centralization measures (*DC*, *CC* and *BC*) (Table 2). Likewise, there was no effect of the alien on link density (*d*) (Table 2) or network diameter (*D*) (*Z* = 1.414, *P* = 0.157). Such results lead us to reject our fifth prediction and conclude that centrality and centralization measures were not affected by the aliens. A consistent pattern of heterogeneity of links in the network (i.e. a disassortative network) was found in ten out of the 12 networks: *dc* of a species was negatively correlated to average *dc* of its nearest neighbours (Table 1). The only locality at which we detected an effect of *Opuntia* was Ravine: the non-invaded site showed a disassortative pattern (*R^2^* = 0.68, *P* = 0.003), whereas links were more homogeneous in the invaded site (*R^2^* = 0.25, *P* = 0.12).

### Temporal and spatial variability

The analyses comparing species and network parameters between 2005 and 2006 gave rather consistent results. No significant effect of year was found for any of the parameters and this was consistent at the two localities studied during the two years (Tables 2 and 3). Thus, alien effects on the pollination networks seemed to be temporally constant. By contrast, there was high spatial variability for the species-level parameters, which supports the idea of a context-dependency of network parameters and alien effects.

## Discussion

Our results confirm that a single alien plant species has the capacity to modify parameters describing the patterns of interactions between native plants and pollinators and between native plants sharing pollinators. Two previous studies have evaluated the impact of alien species on some parameters describing network structure. First, Lopezaraiza-Mikel et al. [7] reported that sites invaded by aliens had higher richness and abundance of flower visitors and higher flower visitation rates than non-invaded ones. More recently, by analyzing ten pairs of plant-pollinator networks with different densities of alien species, Aizen et al. [3] found evidence of a decline in linkage among native species (though not in network connectance) in highly invaded networks, attributing it to a rewiring of links from generalist native species to super-generalist alien species during the invasion process. Thus, our study supports some of the predictions about the possible changes that native plant communities may experience after the introduction of an alien species. Nonetheless, we are still far from being able to predict the extent and direction of such modifications along the invasion process, given the spatio-temporal variability usually found in insect and plant abundances, and due to the intrinsic characteristics of each community [18], [21], [22].

The effect of an alien plant species altering patterns of plant-plant interactions mediated through pollinators has been little examined so far. Changes in such interactions may encompass, for instance, the appearance of new links between unconnected plants. These new links, in turn, may translate into interspecific interference, such as the deposition of heterospecific pollen hampering conspecific pollen germination [23, and references therein]. Likewise, the introduction of an alien may lead to the disruption of previous interactions between native, pollinator-sharing plant species, with possible negative consequences [4]. Thus, we argue that the information obtained from uni-modal networks can be as valuable as that coming from the commonly analyzed bi-modal networks and thus, both are worth studying.

Below, we discuss our findings from these two network perspectives.

### Plant-pollinator networks

As previously found [1], [7], [13], [21], our study shows how an alien plant integrates into a pollination network by interacting with the most generalist insects. This was the case in both archipelagos, despite the fact that *Opuntia* acted as a specialist in Tenerife and as a hub in Menorca. Moreover, our findings also support the argument that endemic super-generalists, such as the bumblebee *B. canariensis*, play an important role as integrators of aliens into pollinator networks [1]. *Bombus canariensis*, together with *Apis mellifera* (considered as introduced in the Canary Islands), were the most frequent visitors of *O. dillenii* in Tenerife, whereas *O. maxima* was visited by a high number of the Menorcan pollinators in the native community. The interaction between *Opuntia dillenii* and the honeybee, specifically, constitutes an example of the beginning of a potential invasional meltdown [24]–[26] on islands, where an alien invasive species facilitates the integration and the expansion of another invader in the system.

As expected, no effect of the alien on connectance was observed. At least in Menorca, links in the community may be transferred from generalist native to super-generalist alien during the invasion [3], but, probably most often, aliens simply establish new links to native pollinators without any accompanying loss of links among natives. A reduction in the number of visits and in pollinator efficiency might still occur, but these effects have to be explored by other approaches and by using quantitative network parameters.

At species level, we found that insects in the invaded sites interacted with a lower number of plants. In other words, the alien appeared to usurp pollinators to natives (at least in Menorca where it behaves as a generalist). Such usurpation of links might have important implications, especially for the reproductive success of plants that may strongly depend upon a particular pollinator that is ‘more attracted’ by the alien when it is present [3], [23]. Such implications might be not only ecological but also evolutionary, as selective pressures on both the plant and the pollinator no longer interacting (or interacting much less) would probably be different between the invaded and the non-invaded site. However, differences in the plant linkage level were not significant; i.e. overall, plants were visited by similar numbers of insect species in invaded and non-invaded sites. This apparent contradiction (changes in *L_m_* but not in *L_n_*) could be attributed to the lower number of plants compared to insects in the pollination networks and to the higher heterogeneity (and thus greater standard deviation) in the linkage levels of the former. Comparing islands, we found higher linkage level for pollinators in Tenerife than in Menorca, which may be due to the oceanic nature of the former. Insects in oceanic islands tend to be strong generalists [1].

The effect of the invader on nestedness was minor. In general, large networks are more likely to be nested than small ones [5], [27]. This might be the reason why we found nestedness in the larger Menorcan networks. Except for one Canarian locality, relative nestedness showed somewhat higher values in invaded than in non-invaded sites, suggesting that *Opuntia* might slightly contribute to increase nestedness. The mechanism for this, however, would be different between the two *Opuntia* species. On the one hand, *O. dillenii* behaves as a specialist, but its few pollinators also visit the most generalist native plant species -which enhances nestedness-. On the other hand, *O. maxima* is one of the most generalist species, interacting with many pollinators which also visit the most specialist plants, and thus enforcing the nestedness pattern. Working with a related species, *O. stricta*, Bartomeus et al. [28] found also a marginal increase in nestedness at invaded sites in the Iberian Peninsula compared to non-invaded ones. Thus, an alien species may enhance nestedness unless it is a specialist linking to native specialists (which seems very unlikely and never reported) or it destroys the native linkage pattern, for instance by stealing generalist partners from native generalists, i.e. attacking the core of native links. An increase in nestedness of mutualistic networks may increase their robustness [5], [29]. However, this does not preclude aliens from having a negative effect on individual native species, for example, by reducing their number of links (or at least number of visits from pollinators), which may translate into a lower reproductive plant success [23], [30]. As pointed out by Aizen et al. [3], super-generalist aliens could actually become central nodes of highly invaded webs, increasing nestedness and contributing to the persistence of many species [5], but at the expense of greatly modifying network architecture during the invasion process.

### Plant-plant networks

As previously mentioned, uni-modal networks constitute an appropriate tool, and complementary to the bi-modal networks, when studying invasions, helping us to identify the mechanisms by which species (in our case, plants) interact positively (facilitation) or negatively (competition) with others by means of their shared pollinators. To assess such competition or facilitation processes, we can later design experiments focusing on particular groups of native species [30]–[34].

We did not detect any impact of the alien on the parameters describing the topology of the uni-modal networks. In the case of link density, this was unexpected, at least in Menorca where *Opuntia* acted as a hub and pollinators visiting it might lead to the establishment of new interactions between native plants and the alien. That link density was unaffected by the alien was probably related to the lower pollinator linkage levels found in the invaded bi-modal networks; that is, rather than allowing the establishment of new interactions between insects and native plants in the invaded sites, aliens usurped links among them.

Despite finding no significant impact of the invader on network parameters, we can foresee, by looking at Figure 1, the possible competition that may exist between the alien and particular native species, worth exploring in future studies. We found that these networks behave as ‘small-worlds’ [6, and references therein], and that the presence of the alien did not alter such properties. Overall, plant uni-modal networks show a disassortative pattern, i.e. species with more connections tend to connect to species with fewer connections. This network pattern has been proposed to provide robustness against perturbations [14]. *Opuntia* remained peripheral to network topology by linking to generalist natives, and this is probably why native network properties were not affected at least in one of the islands. Once a site is intensively invaded, however, the alien may destroy such disassortativeness, making the invaded community less resistant to the entrance of new aliens.

The higher degree values found in Menorca compared to Tenerife indicate that in Menorca, there is a higher number of plants sharing pollinators with other plants, which is probably due to the larger network size in this island and the generalism of the flower visitors. Moreover, the higher closeness centrality in Menorca implies that such plants are more closely connected (with shorter paths between them) in this island than in Tenerife, which we attribute to the presence of *O. maxima*, another generalist species in the community which keeps the network more cohesive. Finally, the centralization measures confirmed, too, that the plant uni-modal networks were very robust against the impact of an invader.

### Temporal and spatial variability

The design of our study (4 sites, 2 years) allowed us to test for temporal and spatial variability of the observed patterns by comparing pairs of sites (invaded *vs.* non-invaded). In general, no significant temporal variation was observed. Although species composition of the communities varied over time (mainly the pollinators), parameters describing the topology of the networks stayed relatively constant, a result that agrees with recent studies focusing on year-to-year variation in pollination networks [17]–[19]).

Nevertheless, in both islands, *Opuntia* showed a higher betweenness centrality in 2006 (the driest year and that with less flowering species in late spring) than in 2005, indicating that this alien kept the network more cohesive during the second year. In the Canarian locality of Ravine, the significantly higher pollinator linkage level observed in 2005 compared to 2006 at the invaded site might also be attributed to the drier weather during the second year (2005: mean spring temperature (MST) = 18.9°C, total spring precipitation (TSP) = 104.9 mm; 2006: MST = 19.9°C, TSP = 41.0 mm). In 2006, there were less flowering species or these had fewer flowers per plant than in 2005. However, an effect of such temporal variation might not be observed at all sites: in Llimpa (Menorca), 2006 was also much drier than in 2005 (2005: MST = 16.5°C, TSP = 131.5 mm; 2006: MST = 17.3°C, TSP = 45.9 mm) but linkage levels were similar (both at the invaded and non-invaded sites) in the two years, despite the fact that number of flowering species and flower abundance also were lower. Hence, the temporal effects may be context-dependent and certainly can only be understood with longer time series. The spatial variation in network parameters supports this idea of context-dependency and we conclude that even relatively simple island systems can behave rather differently.

We demonstrate that the network analytical approach is valid in evaluating and predicting the ways in which aliens may influence native communities. Our results also show that the analyses of both bi-modal and uni-modal networks produce complementary information about the overall complexity of species interactions within and among communities. Thus, we recommend the use of network analysis in the study of biological invasions.

## Materials and Methods

### Study sites

**Figure 2:**
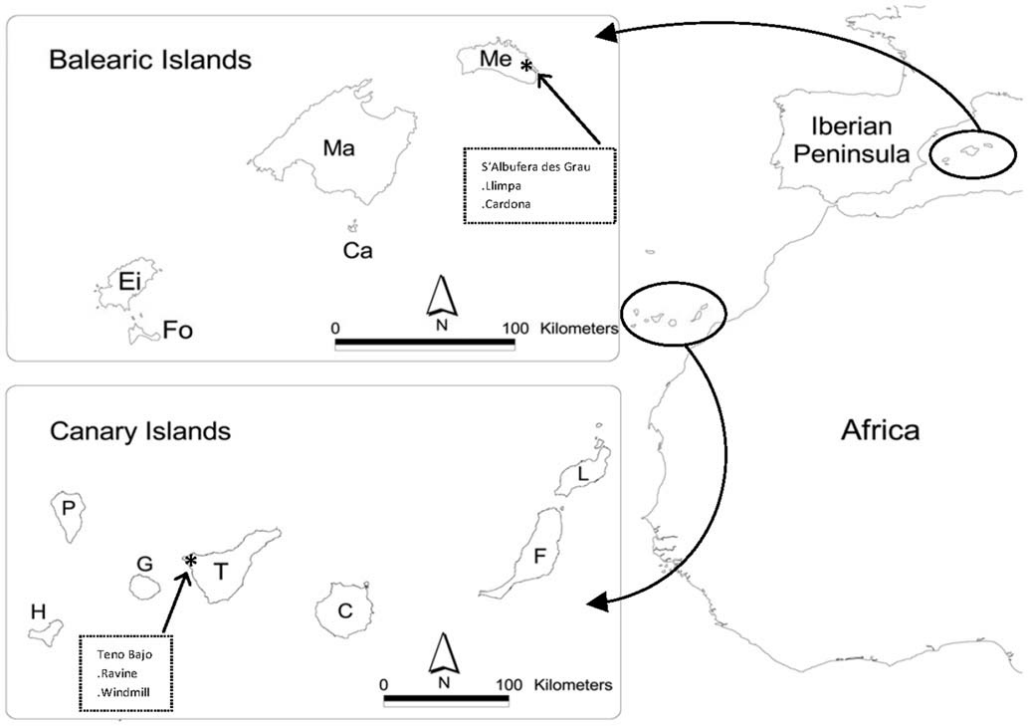
Location of the study sites in the Canary and the Balearic Islands. Ravine and Windmill localities are in the *Teno Bajo* area (Tenerife) and Cardona and Llimpa localities are in *S'Albufera des Grau* Natural Park area (Menorca).

The study sites are located in Tenerife, the largest (2034 km^2^) of the Canary Islands and in Menorca, the second largest (702 km^2^) of the Balearic Islands (Figure 2). In Tenerife, fieldwork was carried out in *Teno Bajo* (28°21′18.83″N; 16°54′19.13″W), in the northwest, a strongly eroded massif with deep seaward-trending ravines (see [35] for a detailed description of the site). The climate is xeric, with an annual mean T of *c.* 21°C and a mean annual rainfall of 200–300 mm. The study sites are in rocky, coastal habitats, with a high number of endemic plants. The vegetation consists of low and sparse xerophytic shrubs, the predominant species being *Rubia fruticosa* (Rubiaceae), *Plocama pendula* (Rubiaceae), *Euphorbia* spp. (Euphorbiaceae), *Withania aristata* (Solanaceae), *Periploca laevigata* (Asclepiadaceae) and the alien *Opuntia dillenii* (Cactaceae) [36]. Here, this alien has a density of *c.* 1200 indiv./ha in the most invaded parts. Data on pollination networks were obtained at two localities with similar vegetation (60% spp. shared), named Ravine and Windmill, separated by a distance of 1 km.

In Menorca, fieldwork was carried out near S'Albufera d'Es Grau Natural Park (39°56′24.98″N; 4°15′6.31″E), also near the coast. The climate here is typically Mediterranean, with an annual mean T of *c.* 17°C and a mean annual rainfall of 573 mm. The habitat is Mediterranean shrubland mixed with abandoned fields, with vegetation dominated by *Pistacia lentiscus* (Anacardiaceae), *Olea europaea* (Oleaceae), *Phyllirea* spp. (Oleaceae), *Ampelodesmos mauritanica* (Poaceae) and herbaceous species such as *Daucus carota* (Apiaceae), *Foeniculum vulgare* (Apiaceae) and *Scolymus hispanicus* (Asteraceae) [37]. The alien *Opuntia maxima* (Cactaceae) attains a high density at some localities (*c.* 200 indiv./ha), mainly around old houses. Data on the pollination networks of this area were also gathered at two localities, named Llimpa and Cardona, *c.* 2.8 km apart and sharing, as in Tenerife, 60% of the plant species.

### The alien plant species

*Opuntia* (Cactaceae), native to the American continent, consists of >200 species, non-columnar and capable of CAM photosynthesis. These cacti were first introduced to Europe by Spanish conquerors towards the end of the 15^th^ century for various purposes, such as human consumption, livestock, foraging, fencing, the production of a red dye that was obtained from a cochineal insect parasite (*Dactylopius coccus*), and as ornamentals [38]. Previous studies in the Iberian Peninsula show that disturbed areas are at greatest risk of invasion by *Opuntia* [39]. Our study species, *O. dillenii* and *O. maxima*, are completely naturalized in the Canaries and the Balearics, respectively. Vegetative multiplication by cladodes and sexual reproduction are common in these species. The flowers are yellow (occasionally orange in *O. maxima*), hermaphroditic, actinomorphic, epigynous, 5–6 cm (*O. dilleniii*) and 7–8 cm (*O. maxima*) in diameter and rich in pollen and nectar [40].

### Study design

Fieldwork took place during the *Opuntia* flowering periods (June) in 2005 and 2006. At this time, the spring bloom of most native species has ceased and, thus, only late-flowering native species overlap with the alien. At each locality on each island, we selected an *Opuntia*-invaded and a non-invaded site, of *c.* 2200 m^2^ in size, i.e. eight sites in total. The distance chosen (300–500 m) between the two sites in each locality was a compromise between having similar and comparable altitude, climate, soil and vegetation composition and density, while simultaneously preventing mixing of insect communities between the two sites. In a previous pairwise comparison, no significant differences were found in animal/plant species ratio (A/P) at each site (*Z* = 0.314, *P* = 0.753). Invaded and non-invaded sites shared *c.* 66% of all plant species. At each site on sunny and non-windy days, we made randomized insect censuses on individuals of each flowering species. A mean of five days was spent at each site. When a plant individual was too large to allow all flowers to be observed simultaneously for insect visitation, we selected an area of 1 m^2^ of plant ‘surface’ for census. Insect visits to a plant were recorded from a distance of 1 m to minimize interference. Censusing began one minute after arriving at a plant and lasted for three minutes. Every flying insect touching a flower or a flower head was noted. Some individuals of each ‘morpho-species’ were caught for later identification. We follow *Fauna Europaea* (http://www.faunaeur.org) and *Herbari Virtual del Mediterrani Occidental* (http://herbarivirtual.uib.es/cat-uib/index.html) for species in Menorca and [41] for species in Tenerife. With our randomized design, the most common flowering plant species were the most observed. However, all plant species at a site received >10 observations.

In June 2005, a total of 847 and 767 of insect censuses were made in Tenerife and Menorca, respectively. By contrast, in June 2006, we made insect census only at one of the two localities in each archipelago: Ravine (Tenerife) and Llimpa (Menorca). This allowed us to analyze spatial variation within-year (four sites in 2005 in each archipelago) as well as temporal dynamics between years in two sites at each archipelago. In 2006, total censuses were 450 and 431 in Tenerife and Menorca, respectively.

### Bi-modal pollination network

This kind of network is described by the parameters *A*, *P*, *M*, *S*, *I* and *C* (Table 4). We used the ratio *C*/*M* to compare networks, given that *C* is negatively correlated with network size [42]. The same correction was done for those parameters influenced or correlated with network size (as linkage levels for plants and pollinator species) and similar results were obtained when correcting for number of species in the communities instead of network size.

**Table 4:** Parameters included in the analyses.

Property		Definition
**2-mode network**		**network linking two groups of communities (plants and pollinators)**
P	plant community size	n° plants
A	pollinator community size	n° pollinators
M	network size	A*P
S	n° species	A+P
I	link number	n° links between A and P
C	connectance	I/(A*P)
L_n_	plant linkage level	n° links between plant species n and the pollinator community
L_m_	pollinator linkage level	n° links between pollinator species m and the plant community
MT	matrix temperature	matrix temperature
MN	matrix nestedness	(100−MT)/100
N*	relative nestedness	N* = (MN−N_R_)/N_R_
BR	Brualdi & Sanderson index	number of discrepancies (absences or presence) that must be erased to produce a perfectly nested matrix
**1-mode network**		**conections among plants in the community**
N	community size	A or P
m	link number	n° links between N
d	link density	2*m/(N*(N−1))
<l>	characteristic path length	n° steps (i.e. links) along the shortest path between two species, averaged over all pairs of species
D	network diameter	the longest of all shortest l of any species pair in the network
c_i_	clustering coefficient	density of links within the neighbourhood of species i
dc	degree centrality	n° links between a species and all other species in the network (named *k* in other studies)
cc	closeness centrality	number of other vertices divided by the sum of all distances between the vertex and all others
bc	betweenness centrality	proportion of all geodesics between pairs of vertices of other vertices that include this vertex
DC	degree centralization	variation in the degrees divided by the maximum degree variation which is possible in a network of the same size
CC	closeness centralization	variation in the closeness centrality divided by the maximum variation in a network of the same size
BC	betweenness centralization	variation in the betweenness centrality divided by the maximum variation in a network of the same size
CCO	connectivity correlation	dc vs. mean dc

A matrix is nested if the links of a species are a subset of the links of more connected species [29, and references therein]. Nestedness temperature (*MT*) is a measure of the degree of nestedness, ranging from 0° (perfectly nested pattern) to 100° (complete checkerboard pattern). Degree of nestedness can be also be expressed as *MN* = (100−*MT*)/100, with values ranging from 0 (checkerboard) to 1 (perfectly nested). Different nestedness algorithms are available. Here, we used *Aninhado* [43] and *Binmatnest* [27]. We used two null models: in model I, each cell in the matrix has the same probability of being occupied [44], whereas in model II that probability depends on the total number of links of the column and the row defining the cell position [5]. *N*_R_ is the mean nestedness of the random runs. *Aninhado* provides an *idiosyncratic temperature* for each species, telling us to what extent each species contributes to network temperature. More recent software, *Nestedness*, is provided by Ulrich and Gotelli [20] who argue that temperature is not an appropriate measure of nestedness, recommending instead the discrepancy index *BR* [45]. We calculated both *BR* and *MT* with the *Nestedness* program, using null model I and the fixed-fixed null model [20], which is close to null model II above. A total of 1000 Monte Carlo randomizations were used in runs with *Aninhado* and *Binmatnest*, and 100 with *Nestedness* [20]. We used *relative nestedness*, N* = (*MN*−*N*_R_)/*N*_R_ to compare across networks, controlling for variation in number of species and links [5].

### Uni-modal pollination network

For each uni-modal network, we calculated the following species properties: degree centrality (*dc*), closeness centrality *(cc*), and betweenness centrality (*bc*) (Table 4) [16]. The former refers to the number of links per plant, while the other two have a maximum value of 1, as they are proportions. Closeness centrality is a measure of how closely a species is connected to other species in the network, based on the shortest paths between them. On the other hand, betweenness centrality of a particular species is the proportion of paths between any combination of two species in the network that pass through that particular species. We further obtained values of two more parameters widely used in uni-modal networks to describe their topology: clustering coefficient (*c*) and path length (<*l*>). The former is the proportion of realized links among the species' neighbours, whereas the latter is the shortest path between two species measured in number of links and averaged over all pairs of species in the community, being a network level parameter (Table 4) [15].

Also at the network level, we obtained values of the three components of the centralization parameter: degree centralization (*DC*), closeness centralization (*CC*) and betweenness centralization (*BC*) [16]. A network with high *DC* has a core of one or a few highly connected species, and a periphery of species more loosely connected to the network. Such a network is vulnerable to the extinction of a core species, but robust against removal of the peripheral species. *CC* is the variation in the *cc* of vertices divided by the maximum variation in *cc* scores possible in a network of the same size. In social sciences, this parameter is used to know how fast information spreads in a network of people; in mutualistic networks, such “information” might for instance be the effect of a disturbance, such as an invasion. Lastly, *BC* is the variation in the *bc* of vertices divided by the maximum variation in *bc* scores possible in a network of the same size and networks with high *BC* have a few species with very high *bc*. At this network level, we also obtained two other parameters previously used in uni-modal pollination networks [6] and useful to describe network structure: link density (*d*), which measures how connected the network is (it is equivalent to the connectance parameter (*C*) in bi-modal networks), and network diameter (*D*), which is the longest of all shortest *l* of any species pair in the network (Table 4).

Furthermore, the correlation between *dc* of a species and the average *dc* of its nearest neighbours was also examined. A negative correlation implies a heterogeneous link density in the network, which is called disassortative, i.e. species with more connections tend to connect to species with fewer connections [14].

### Statistical analysis

Parameters describing network topology were calculated using the software Pajek v. 1.18. [16]. Correlation, linear and non-linear regression analyses were performed using both SPSS v. 15.0 for Windows and *R* v. 2.5.1. For network level parameters (only one value per site), we performed Wilcoxon's tests to compare invaded *vs.* non-invaded sites using pair of sites observed in 2005 and to compare sites between years. For parameters obtained at species level, we used generalized linear models (GLM) including island, locality (nested within island) and site (invaded *vs.* non-invaded) as fixed factors. In order to compare such parameters between years, we again performed GLMs but this time including site and year (2005 vs. 2006) as fixed factors. We use normal or gamma distributions (when a transformation does not normalize the variable) depending on the best fit for each parameter.
